# Supplementary material for: Aβ initiates brain hypometabolism, network dysfunction and behavioral abnormalities via NOX2-induced oxidative stress in mice
Source: Commun Biol. 2021 Sep 9;4:1054. doi: 10.1038/s42003-021-02551-x (PMC8429759; doi:10.1038/s42003-021-02551-x)
Supplement: Supplementary file 2 — Supplementary Information [file 42003_2021_2551_MOESM2_ESM.pdf]

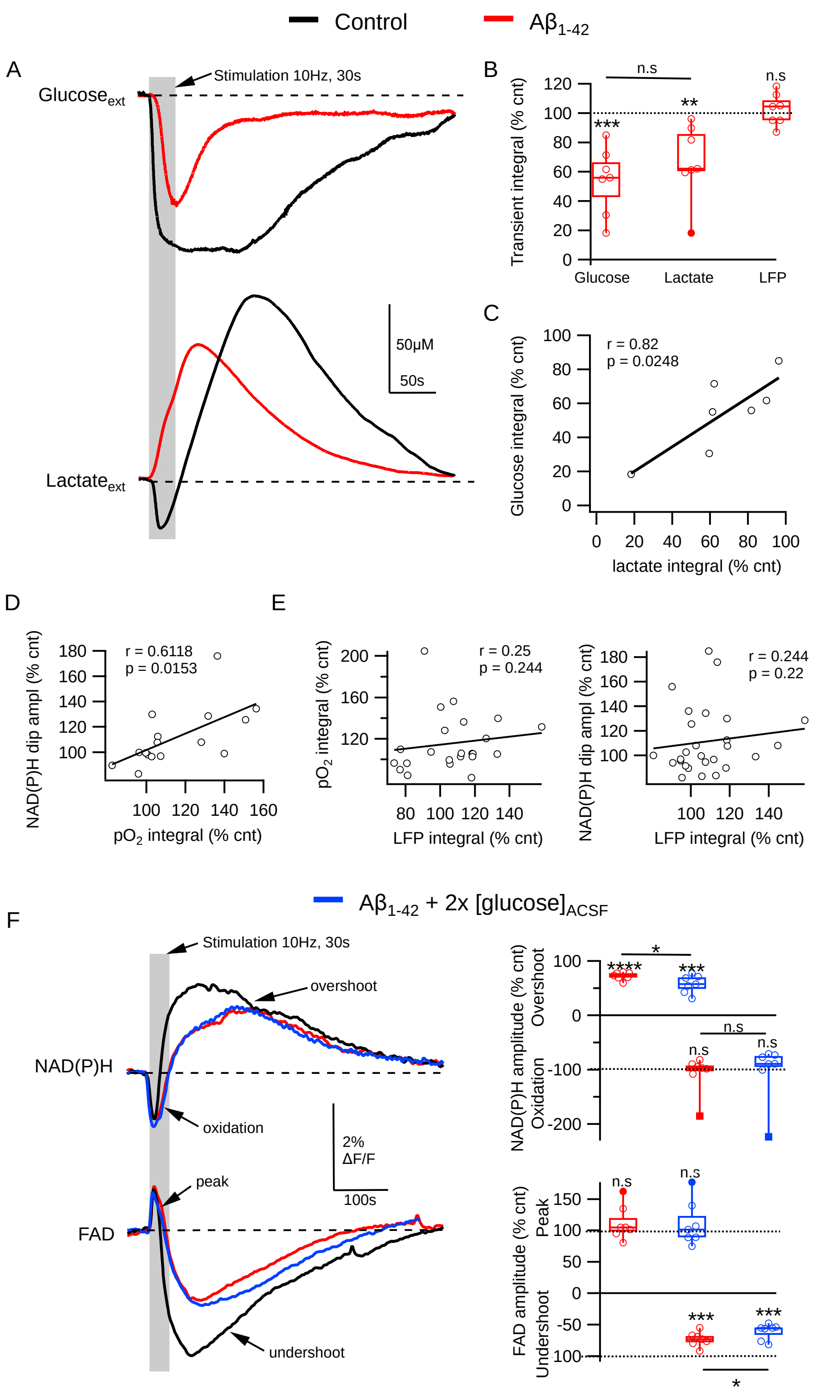

Supplementary Figure 1. ***Multiple effects of Aβ toxicity on glucose metabolism and glycolysis.*** **A**. Example traces from a single experiment in brain slice with activity-driven extracellular glucose and lactate transients measured simultaneously in CA1 in control (black) and following 40 minutes of A$\beta_{1\text{-}42}$ application (red). **B**. Summary of all such experiments. **C**. Decrease in activity-driven lactate release is significantly correlated with reduction in glucose uptake under Aβ. **D.** Aβ-induced increase in NAD(P)H oxidation phase amplitude is positively correlated with the parallel increase in oxygen consumption. **E.** Changes in metabolic transients are not correlated with changes in network response seen as LFP integrals. **F.** Increasing extracellular glucose concentration has no effect on Aβ-induced disruption of glycolysis. Left, Example traces from a single experiment in brain slices with activity-driven NAD(P)H and FAD fluorescence transients measured simultaneously in control (black), following 40 minutes of A$\beta_{1\text{-}42}$ application (red), and after subsequent doubling of ACSF glucose concentration (blue). Note that Aβ-induced reduction in NAD(P)H overshoot is paralleled by a reduction in FAD undershoot, confirming inhibited glycolysis. Right, summary of all such experiments. Data are presented as box plots with min-max whiskers and Tukey quartile method. *$p < 0.05$, **$p < 0.01$, ***$p < 0.001$, ****$p < 0.0001$.

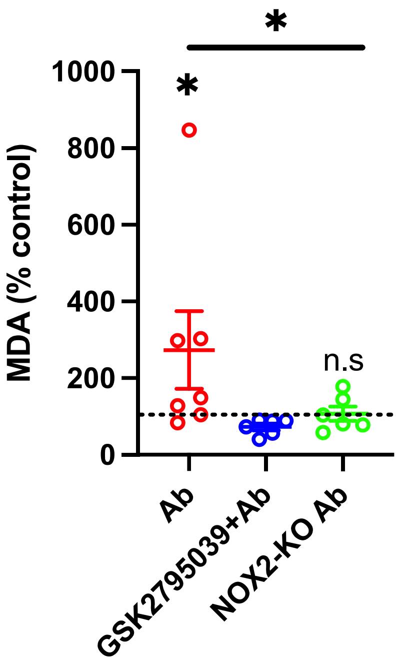

Supplementary Figure 2. ***Aβ induces lipid peroxidation via activation of NOX2.*** Individual brain slice MDA levels, normalized to corresponding non-Aβ controls. Red: WT slices exposed to 400nM fibrillar $A\beta_{1-42}$ for one hour; Blue: WT co-treated with $A\beta_{1-42}$ and GSK2795039; and Green: Brain slices from NOX2-deficient ($Cybb^{tm1din}$/J) mice exposed to $A\beta_{1-42}$. *$p<0.05$. Error bars are ±SEM.

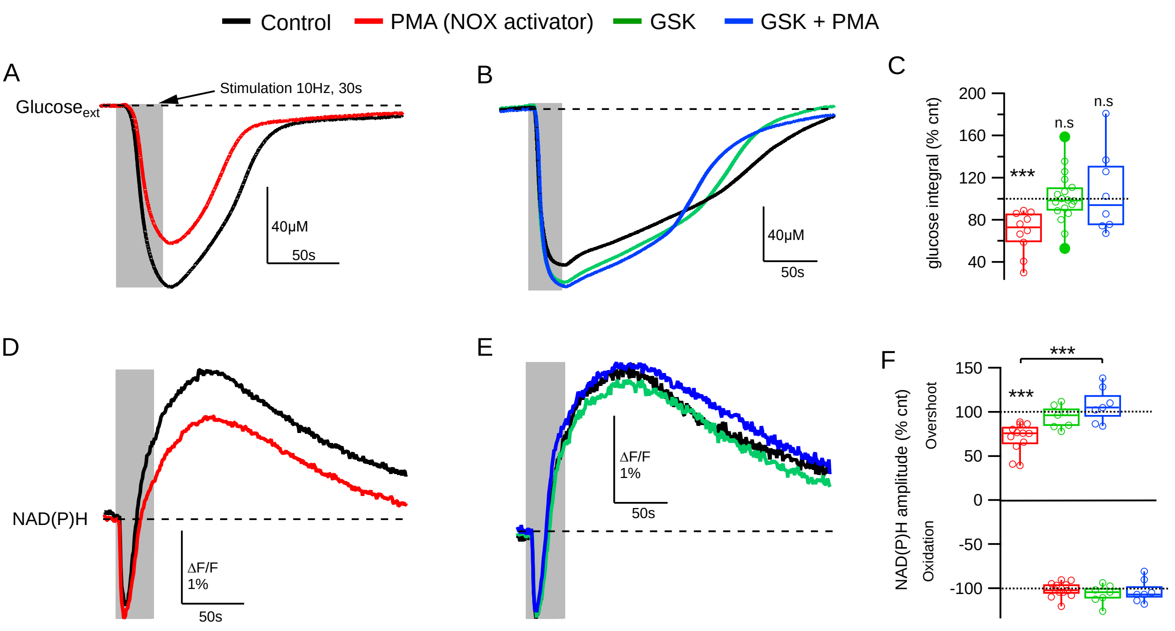

Supplementary Figure 3. ***Direct activation of NOX by PMA reproduces the effect of*** **Aβ1-42** ***on glucose utilization*.** **A.** Application of NOX activator PMA (100nM) reduces glucose uptake; average traces from a single experiment showing extracellular glucose transients in CA1 pyramidal cell layer in response to a 10Hz, 30s stimulation of Schaffer collaterals (grey) in control (black) and following 40min of PMA application (red) **B.** GSK2795039 prevents the effects of PMA on activity-driven glycolysis. Example traces from a single experiment showing glucose transients in control (black), after application of GSK2795039 (green), and following GSK2795039+PMA application (blue). **C**. Summary graph of all corresponding glucose transient integral values in A-B. **D**. PMA reduces glycolysis: NAD(P)H autofluorescence traces from a single experiment in control (black) and after application of PMA (red). **E**. PMA effect on glycolysis is blocked by GSK2795039: NAD(P)H autofluorescence traces from a single experiment in control (black), after application of GSK2795039 (green), and following GSK2795039+PMA application (blue). **F**. Summary graph of all corresponding NAD(P)H amplitudes in D-E. Data are presented as box plots with min-max whiskers and Tukey quartile method. ***$p<0.001$

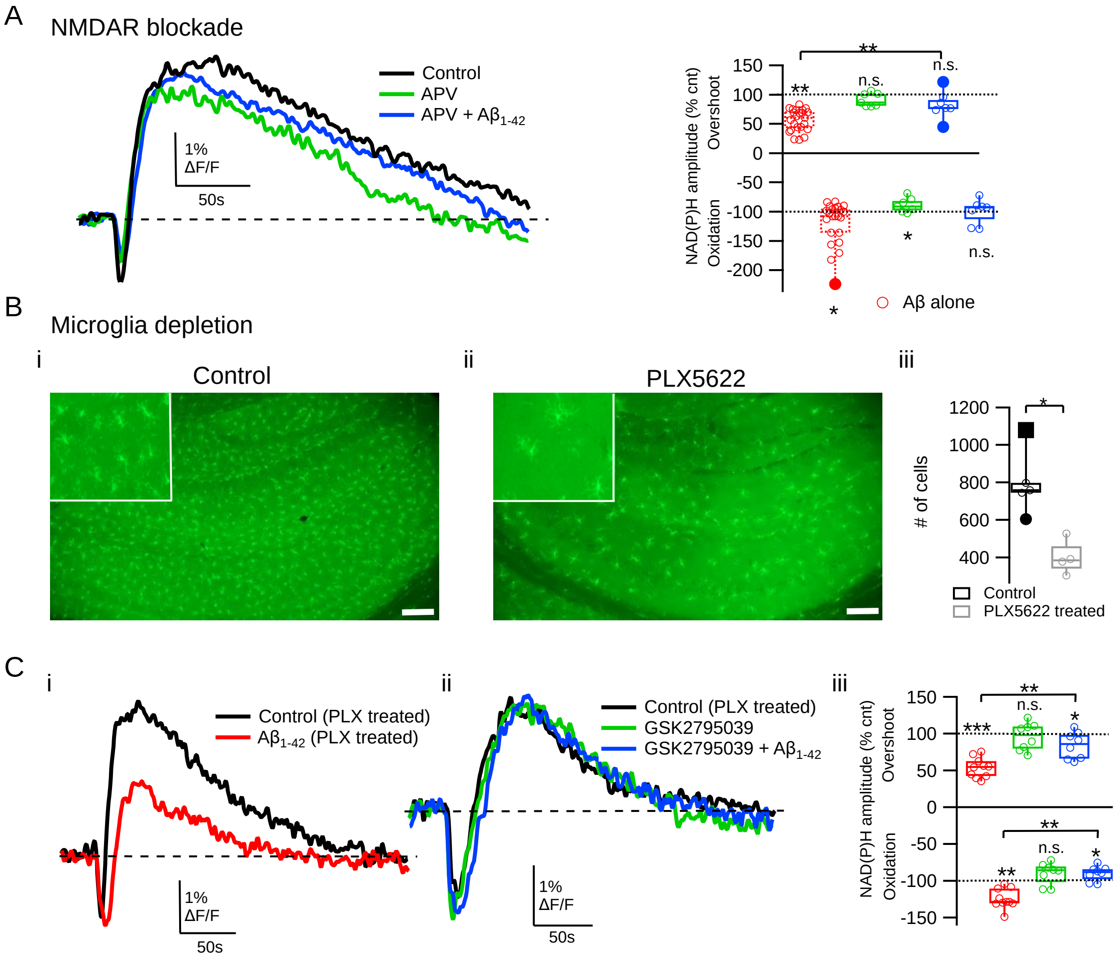

Supplementary Figure 4. ***Locus of NOX2 expression behind Aβ-induced glucose hypometabolism.***
A. NMDA receptor blockade by APV prevents Aβ effects on glycolysis, suggesting neuronal NOX contribution. Left, example traces from a single experiment showing activity-induced NAD(P)H autofluorescence transients in control (black), following APV application (green), and after subsequent application of $A\beta_{1\text{-}42}$ (blue). Right, the summary plot of NAD(P)H oxidation and overshoot amplitudes; red markers indicate Aβ values from Figure 1 for comparison. **B, C.** Microglial depletion does not prevent Aβ effect on glucose utilization. **B.** Sample images of *ex-vivo* hippocampal microsections stained for Iba1 antibody from mice following 1 week of control (i) and PLX5622-containing diet (ii). Scale bar = 100µm iii*)* Iba1+ cell counts across all slices. **C.** Hippocampal slices from PLX5622-treated mice are not protected from Aβ toxicity on glucose utilization. *i)* traces from a single experiment showing activity-induced NAD(P)H autofluorescence transients in control (black) and following application of $A\beta_{1\text{-}42}$ (red) in slices from microglia-depleted brains. *ii)* NOX2 blockade in microglia-depleted slices prevents Aβ effects on glycolysis. *iii)* summary plot of corresponding NAD(P)H transient amplitudes. Data are presented as box plots with min-max whiskers and Tukey quartile method. *p<0.05, **p<0.01, ***p<0.001.
