# Supplementary material for: Aβ initiates brain hypometabolism, network dysfunction and behavioral abnormalities via NOX2-induced oxidative stress in mice
Source: Commun Biol. 2021 Sep 9;4:1054. doi: 10.1038/s42003-021-02551-x (PMC8429759; doi:10.1038/s42003-021-02551-x)
Supplement: Supplementary file 3 — Description of Additional Supplementary Files [file 42003_2021_2551_MOESM3_ESM.docx]

**Description of Additional Supplementary Files**

**File name:** Supplementary Movie 1

**Description:** Social Interaction test on three mouse groups (vehicle, A-beta, and A-beta+ GSK2795039) to demonstrate the grouping and fighting episodes. The video is 15 representative minutes, with first grouping episodes highlighted at 0:13 and fighting at 4:30.

**File name:** Supplementary Data 1

**Description:** Data used in main Figures.

**File name:** Supplementary Data 2

**Description:** Data used in Supplementary Figures.
