# Supplementary material for: Aβ initiates brain hypometabolism, network dysfunction and behavioral abnormalities via NOX2-induced oxidative stress in mice
Source: Commun Biol. 2021 Sep 9;4:1054. doi: 10.1038/s42003-021-02551-x (PMC8429759; doi:10.1038/s42003-021-02551-x)
Supplement: Supplementary file 7 — Reporting Summary [file 42003_2021_2551_MOESM7_ESM.pdf]

nature research

Corresponding author(s): Misha Zilberter

Last updated by author(s): Oct 5, 2020

Reporting Summary

Nature Research wishes to improve the reproducibility of the work that we publish. This form provides structure for consistency and transparency in reporting. For further information on Nature Research policies, see our Editorial Policies and the Editorial Policy Checklist.

Statistics

For all statistical analyses, confirm that the following items are present in the figure legend, table legend, main text, or Methods section.

n/a	Confirmed	
☐	☒	The exact sample size (*n*) for each experimental group/condition, given as a discrete number and unit of measurement
☐	☒	A statement on whether measurements were taken from distinct samples or whether the same sample was measured repeatedly
☐	☒	The statistical test(s) used AND whether they are one- or two-sided<br>*Only common tests should be described solely by name; describe more complex techniques in the Methods section.*
☐	☒	A description of all covariates tested
☐	☒	A description of any assumptions or corrections, such as tests of normality and adjustment for multiple comparisons
☐	☒	A full description of the statistical parameters including central tendency (e.g. means) or other basic estimates (e.g. regression coefficient) AND variation (e.g. standard deviation) or associated estimates of uncertainty (e.g. confidence intervals)
☐	☒	For null hypothesis testing, the test statistic (e.g. *F*, *t*, *r*) with confidence intervals, effect sizes, degrees of freedom and *P* value noted<br>*Give P values as exact values whenever suitable.*
☒	☐	For Bayesian analysis, information on the choice of priors and Markov chain Monte Carlo settings
☒	☐	For hierarchical and complex designs, identification of the appropriate level for tests and full reporting of outcomes
☒	☐	Estimates of effect sizes (e.g. Cohen's *d*, Pearson's *r*), indicating how they were calculated

Our web collection on statistics for biologists contains articles on many of the points above.

Software and code

Policy information about availability of computer code

Data collection	PClamp v10 and 11 (Molecular Devices, USA), Patchmaster (HEKA Elektronik GmbH, Germany), WinFluor (University of Strathclyde, Glasgow, UK)
Data analysis	Igor Pro 8 (WaveMetrics Inc, USA); ImageJ.

For manuscripts utilizing custom algorithms or software that are central to the research but not yet described in published literature, software must be made available to editors and reviewers. We strongly encourage code deposition in a community repository (e.g. GitHub). See the Nature Research guidelines for submitting code & software for further information.

Data

Policy information about availability of data

All manuscripts must include a data availability statement. This statement should provide the following information, where applicable:

- Accession codes, unique identifiers, or web links for publicly available datasets
- A list of figures that have associated raw data
- A description of any restrictions on data availability

All data associated with this study are available in the main text or the supplementary materials.

Field-specific reporting

Life sciences study design

All studies must disclose on these points even when the disclosure is negative.

Sample size	Sample sizes were estimated based on pilot and prior similar studies showing significance
Data exclusions	No data was excluded in this study
Replication	Beta-amyloid effect was highly reproducible, as was the inhibitory effect of GSK2795039. Results were successfully replicated on three different mouse strains.
Randomization	Mice were randomly allocated to groups for all behavioral and electrophysiological studies.
Blinding	No blinding was possible during data collection for most of the experiments due to their paired (wash-in/i.c.v. injection of beta-amyloid and/or inhibitors) design. Analysis was not performed blinded.

Reporting for specific materials, systems and methods

We require information from authors about some types of materials, experimental systems and methods used in many studies. Here, indicate whether each material, system or method listed is relevant to your study. If you are not sure if a list item applies to your research, read the appropriate section before selecting a response.

Materials & experimental systems

n/a	Involved in the study	
☐	☒	Antibodies
☒	☐	Eukaryotic cell lines
☒	☐	Palaeontology and archaeology
☐	☒	Animals and other organisms
☒	☐	Human research participants
☒	☐	Clinical data
☒	☐	Dual use research of concern

Methods

n/a	Involved in the study	
☒	☐	ChIP-seq
☒	☐	Flow cytometry
☒	☐	MRI-based neuroimaging

Antibodies

Antibodies used	Anti Iba1, Rabbit (Wako, Japan; Cat.No. 019-19741). Goat anti-Rabbit IgG Secondary Antibody, Alexa Fluor Plus 488 (ThermoPhisher, USA; Cat. No A32731)
Validation	All antibodies were validated by the respective manufacturers.

Animals and other organisms

Policy information about studies involving animals; ARRIVE guidelines recommended for reporting animal research

Laboratory animals	OF1 mice (Charles River Laboratories), C57Bl6/J mice (Jackson Labs), BALB/c mice (Laboratory Animal Nursery "Pushchino", Russia), Cybb_tm1din/J mice (Jackson Labs)
Wild animals	No wild animals were used in this study
Field-collected samples	No field collected samples were used in this study
Ethics oversight	All animal protocols and experimental procedures were approved by the University of California and Gladstone Institutes under IACUC protocol AN176773, the Ethics Committees for Animal Experimentation at the INSERM (protocol #30-03102012), and ITEB RAS.

Note that full information on the approval of the study protocol must also be provided in the manuscript.
